# Draft genome of *Rossellomorea* sp. DUT-2 with Mn(II) oxidation properties isolated from nearshore surface sediments of Liaodong Bay

**DOI:** 10.1128/mra.00441-25

**Published:** 2025-08-11

**Authors:** Junzhi Ma, Wenyiming Xiao, Haixia Pan, Hao Zhou

**Affiliations:** 1 Key Laboratory of Industrial Ecology and Environmental Engineering (Ministry of Education), School of Chemical Engineering, Ocean and Life Sciences, Panjin Campus, Dalian University of Technology, Panjin, China; Rochester Institute of Technology, Rochester, New York, USA

**Keywords:** manganese-oxidizing bacteria, genome, *Rossellomorea*, carbon metabolism, nitrogen metabolism

## Abstract

*Rossellomorea* sp. DUT-2 with Mn(II) oxidation properties was isolated from nearshore surface sediments of Liaodong Bay, China. Genome sequencing was utilized to decipher its genomic function for Mn(II) oxidation and carbon/nitrogen metabolism; the assembly yielded a genome size of 4,378,879 bp with a G + C content of 41.52%.

## ANNOUNCEMENT

Current studies have identified various manganese-oxidizing bacteria within the genera *Bacillus*, *Pseudomonas*, and *Roseobacter*. These bacteria employ diverse enzymes for manganese oxidation, for instance, multicopper oxidases (MCOs) and animal heme peroxidases (AHPs). The MCOs oxidize Mn(II) via two-step one-electron oxidation to form Mn(III) and Mn(IV), while some AHPs, such as *ahpL*, *ahpA*, *ahpB*, and *ahpC*, mediate Mn(II) oxidation by producing superoxide (1, 2).

In this study, we isolated a Mn(II)-oxidizing bacterium, designated as DUT-2, from nearshore sediments of Liaodong Bay located at Liaoning Province, China (121°18′E 40°50′N). DUT-2 was isolated via four generations of serial incubation in modified mineral medium at 30°C. This medium contains yeast extract (0.05 g/L), peptone (0.25 g/L), sodium pyruvate (1.0 g/L), KCl (5.4 g/L), K_2_HPO_4_ (0.30 g/L), CaCl_2_ (0.29 g/L), NaNO_3_ (0.429 g/L), MgSO_4_·7H_2_O (13.4 g/L), MgCl_2_·6H_2_O (11.5 g/L), NaCl (35.0 g/L), and agar (20 g/L if necessary) (3). The MnCl_2_·4H_2_O was supplemented to the culture medium with a final concentration of 100 µM. During the enrichment and purification processes of strain DUT-2, manganese oxidation was confirmed by Leucoberbelin blue solution (4).

The EzBioCloud server was used to identify DUT-2 based on its 16S rRNA sequence (5). The results show that strain DUT-2 belongs to the genus *Rossellomorea* with a 99.32% sequence identity with *Rossellomorea arthrocnemi* EAR8. For the genome sequencing, the bacterial cells were first incubated in the modified mineral medium for 4 days and then the cells were collected by centrifugation. Genomic DNA of strain DUT-2 was extracted by Qiagen DNeasy PowerSoil Pro Kit according to the manufacturer’s instructions. DNA sequencing was performed at Novogene Co., Ltd., Tianjin (China). A paired-end sequencing library was prepared using Illumina Nextera DNA Library Prep Kit, and paired-end 150 bp reads were acquired on an Illumina NovaSeq 6000 platform. FastQC (v0.12.1) was used to evaluate the quality of raw data (1,956.58 Mbp), and Trimmomatic (v0.40) was used to filter low-quality reads and adapter sequences (6, 7). The obtained clean data were assembled through SOAPdenovo2, SPAdes (v4.1.0), and ABySS (v2.3.10) software (8–10). The assembled contigs from these tools were integrated and refined using CISA software (11). The genome sequences were annotated by GeneMarkS software (v4.30) (12). In the genomic analysis, all software was used with default parameters. The final genome assembly of strain DUT-2 comprised 15 contigs, achieving a sequencing coverage of 363×. A total of 15 contigs with G + C content of 41.52% were obtained. Among the 4,607 predicted genes with a genome size of 4,378,879 bp, 104 tRNA, 13 rRNA, including 11 of 5S, 1 of 16S, and 1 of 23S, and 5 sRNA were found in the genome of strain DUT-2.

The taxonomic identification of strain DUT-2 was analyzed by Prokaryotic Genome Annotation Pipeline, and the dDDH (d_4_) value between strain DUT-2 and *R. arthrocnemi* was 25.1%, which is below 70%, suggesting this strain is a potential new species (13). The genes related to Mn(II) oxidation and C, N cycling were annotated by MMseqs2 software, KEGG database, and GapMind (14–16). The annotation results illustrate the existence of two genes (*cotA* and *stKatG*) encoding a manganese-oxidizing protein. Based on KEGG annotation, most of the genes involved in the metabolism of starch, sucrose, and pyruvate were found. Genes associated with nitrogen metabolism, for example, *glnA*, showed the potential of ammonia assimilation. Through GapMind database annotation, several carbon sources, such as glucose, sucrose, fructose, glycerol, L-malate, D-lactate, and L-lactate, could be utilized for the growth of strain DUT-2. Mn-oxidizing genes and associated C/N metabolic pathways identified in strain DUT-2 indicate its utility in Mn bioremediation and sustainable biotechnology.

## Data Availability

The draft genome of *Rossellomorea* sp. DUT-2 has been submitted to the NCBI database with the BioProject and BioSample numbers PRJNA1243322 and SAMN47618716, respectively, and the raw data were deposited into the Sequence Read Archive (SRA) database under the accession number SRR32899801. The 16S rRNA sequence has been submitted to the GenBank (JBNTRC000000000) database under the accession number PV474683.
